# Prone positioning in acute respiratory distress syndrome after abdominal surgery: a multicenter retrospective study: SAPRONADONF (Study of Ards and PRONe position After abDOmiNal surgery in France)

**DOI:** 10.1186/s13613-017-0235-z

**Published:** 2017-02-24

**Authors:** Stéphane Gaudry, Samuel Tuffet, Anne-Claire Lukaszewicz, Christian Laplace, Noémie Zucman, Marc Pocard, Bruno Costaglioli, Simon Msika, Jacques Duranteau, Didier Payen, Didier Dreyfuss, David Hajage, Jean-Damien Ricard

**Affiliations:** 1 Medico-Surgical Intensive Care Unit, Hôpital Louis Mourier, AP-HP, 178 rue des Renouillers, 92700 Colombes, France; 2 Sorbonne Paris Cité, ECEVE UMR 1123, Univ Paris Diderot, 75018 Paris, France; 3 Département d’Anesthésie Réanimation, Hôpital Lariboisière, AP-HP, 75010 Paris, France; 4 UMR U 1160, Université Paris-Diderot Paris 7, 75010 Paris, France; 5 Département d’Anesthésie Réanimation, Hôpital Bicêtre, AP-HP, 94270 Le Kremlin-Bicêtre, France; 6 Hôpital Lariboisière, Chirurgie digestive et cancérologique, AP-HP, 75010 Paris, France; 7 UMR U 965, Université Paris-Diderot Paris 7, 75010 Paris, France; 8 Hôpital Bicêtre, Chirurgie générale et digestive, AP-HP, 94270 Le Kremlin-Bicêtre, France; 9 Hôpital Louis Mourier, Chirurgie digestive, AP-HP, 178 rue des Renouillers, 92700 Colombes, France; 10 UMR 1149, Univ Paris Diderot, Sorbonne Paris Cité, 75018 Paris, France; 11 IAME,UMR 1137, INSERM, 75018 Paris, France; 12 IAME, UMR 1137, Univ Paris Diderot, Sorbonne Paris Cité, 75018 Paris, France; 13 Epidemiology and Clinical Research Department, Hôpital Louis Mourier, AP-HP, 178 rue des Renouillers, 92700 Colombes, France; 14 Service de Réanimation Médicale, 178 rue des Renouillers, 92701 Colombes Cedex, France

**Keywords:** Mechanical ventilation, ARDS, Prone position

## Abstract

**Background:**

The recent demonstration of prone position’s strong benefit on patient survival has rendered proning a major therapeutic intervention in severe ARDS. Uncertainties remain as to whether or not ARDS patients in the postoperative period of abdominal surgery should be turned prone because of the risk of abdominal complications. Our aim was to investigate the prevalence of surgical complications between patients with and without prone position after abdominal surgery.

**Methods:**

This study was a multicenter retrospective cohort of patients with ARDS in a context of recent abdominal surgery. Primary outcome was the number of patients who had at least one surgical complication that could be induced or worsened by prone position. Secondary outcomes included effects of prone position on oxygenation. Data from the prone group were compared with those from the supine group (not having undergone at least a prone position session).

**Results:**

Among 98 patients included, 36 (37%) had at least one prone position session. The rate of surgical complications induced or worsened by prone position did not differ between prone and supine groups [respectively, 14 (39%) vs 27 (44%); *p* = 0.65]. After propensity score application, there was no significant difference between the two groups (OR 0.72 [0.26–2.02], *p* = 0.54). Revision surgery did not differ between the groups. The first prone session significantly increased PaO_2_/FiO_2_ ratio from 95 ± 47 to 189 ± 92 mmHg, *p* < 0.0001.

**Conclusion:**

Prone position of ARDS patients after abdominal surgery was not associated with an increased rate of surgical complication. Intensivists should not refrain from proning these patients.

**Electronic supplementary material:**

The online version of this article (doi:10.1186/s13613-017-0235-z) contains supplementary material, which is available to authorized users.

## Background

Prone positioning has been used for a long time to improve oxygenation in patients with acute respiratory distress syndrome (ARDS) [1]. The different mechanisms explaining its potential benefits include homogenization of ventilation–perfusion mismatch, redistribution of pleural pressure gradient, net alveolar recruitment and more harmonious alveolar inflation [2] and prevention and reduction of ventilator-induced lung injury (VILI) [3, 4]. Until recently, randomized controlled trials (RCT) on prone position failed to show a net benefit on survival [5–8] but had provided cues for a possible benefit among the most severe ARDS patients [9, 10]. Guerin et al. confirmed this hypothesis by demonstrating a strong survival benefit in a large RCT in patients with PaO2/FiO2 ratio <150 mmHg [11]. This resounding demonstration, linked to the fact that prone positioning does not require any special equipment and is not associated with excess side effects (10), suggests all severe ARDS patients should be turned prone in case of refractory hypoxemia [12]. This is true irrespective of the origin (pulmonary or extra-pulmonary) of ARDS, with the exception of trauma patients with spinal instability or unmonitored increased intracranial pressure. Despite these evidences, a recent large international epidemiological study indicates that only 16% of severe ARDS patients are turned prone [13]. Among etiologies of extra-pulmonary origin, those consecutive to abdominal emergencies may constitute an obstacle to the use of prone position and lead to an even smaller percentage than above. In case of severe hypoxemia in the early postoperative period, intensivists could be reluctant to prone patients for fear of repercussions on scars, draining systems and stoma. Cases of midline abdominal wound dehiscence potentially related to prone positioning have been reported [14] but to what extent prone position may induce or worsen postsurgical complications remains unknown. Because prone position is now a major therapeutic intervention in the management of ARDS, it is crucial to determine whether prone position is associated or not with more complications in patients with ARDS after abdominal surgery.

Given that this population represents a minority of patients included the above-mentioned RCT and that there is no questioning of the efficacy of prone position in ARDS, yet another RCT is no desirable (nor feasible) to obtain such determination. We therefore conducted a retrospective, multicenter study to assess the prevalence of surgical complications that could be a priori induced or worsened by prone position among patients developing ARDS after abdominal or pelvic surgery.

## Methods

### Design and ethics

This was a retrospective study performed in three ICUs of Assistance Publique—Hôpitaux de Paris, University Hospitals (Louis Mourier, Lariboisière and Kremlin-Bicêtre) between March 2009 and March 2014 designed to compare the risk of surgical complications that could be a priori induced or worsened by prone position between patients who had at least one prone position session (prone group) and those who remained supine (supine group) after abdominal surgery. Admission of abdominal emergencies in these three ICUs is part of their routine activity. In case of ARDS, decision to prone patients was taken by the ICU physicians and the context of abdominal surgery was not considered as a contraindication.

The study was approved by the Ethics Committee of the French Intensive Care Society (project no. 14-31). We followed the Strengthening the Reporting of Observational Studies in Epidemiology (STROBE) statement guidelines for observational cohort studies [15].

### Study population

Two independent searches on the ICU’s electronic database were performed over the study period, one with the search label “ARDS” (ICD label J80) and the other with “acute respiratory failure” (ICD label J960). The two lists of patients were cross-checked to ensure exhaustibility and verify the final diagnosis of ARDS. Once extracted, medical records were reviewed. Patients were retained in the final analysis if they had an ARDS consistent with Berlin definition [16] (oxygenation criteria: PaO_2_/FiO_2_ <300 mmHg with PEEP or CPAP ≥5 cmH_2_O) in a context of recent (less than 7 days) abdominal surgery. We did not include in the analysis patients who had just had a laparoscopy or who died in the next 48 h following surgery.

The day of inclusion (D0) in the analysis was defined as the day when ARDS occurred.

### Main characteristics of protocol use for the prone positioning placement

During the study period, medical and paramedical teams followed protocol for prone positioning placement. A minimum of four persons were required for the procedure; one of them was placed at the patient’s head to secure the endotracheal tube. Rotation to the left or to the right depended on the location of invasive arterial pressure and central venous lines. The upper limbs were placed alongside the body. Potential pressure points were protected using adhesive pads.

A circular pillow was used to ensure appropriate position of the head and the endotracheal tube. Pillows were placed under the thorax and pelvis in order to limit abdominal pressure.

### Data collection

The data recorded from the files were the following:

*Epidemiological data*: age, sex, weight, body mass index (BMI), chronic obstructive pulmonary disease, ischemic heart disease, systemic hypertension and diabetes.

*Characteristics of ICU severity*: SAPS II [17], septic shock (at D0) defined by Bone’s criteria [18] and catecholamine infusion (at D0).

*Characteristics of ARDS and mechanical ventilation*: lowest PaO_2_/FiO_2_ ratio at D0, highest plateau pressure (Pplat) at D0, lowest tidal volume at D0, highest PEEP at D0, use of adjunctive therapies (including neuromuscular blocking agents, inhaled nitric oxide, prone positioning) and duration of mechanical ventilation. For patients who had at least one prone position session (prone group), data collection included: time between surgery and first prone position session, number and duration of prone position session, PaO_2_/FiO_2_ before (measure on the last arterial blood gas before first prone session) and after (measure on the first arterial blood during the first prone position session) and hemodynamic changes after first prone session. To address this hemodynamic issue, we defined three categories depending on the changes in catecholamine dosage during the first 2 h of the first prone session: i/hemodynamic worsening (defined as increase in catecholamines), ii/hemodynamic improvement (decrease) and iii/hemodynamic stability (no change).

*Characteristics of abdominal surgery*: planned or emergent surgery, delay between surgery and ICU hospitalization, presence of peritonitis (defined according to the International Sepsis Forum Consensus Conference on Definitions of Infection in the Intensive Care Unit [19], type of surgical procedure, number and type of stoma. Not being a routine procedure, intra-abdominal pressure was not systematically measured.

*Postoperative surgical complications*: We defined a priori these complications: scar dehiscence, abdominal compartment syndrome (define as intra-abdominal hypertension >20 mmHg with new organ dysfunction or failure) [20], stoma leakage, stoma necrosis, scar necrosis, wound infection, displacement of a drainage system, removal of a gastro- or jejunostomy feeding tube and digestive fistula. The Clavien–Dindo classification for surgical complications was assessed. However, it was not discriminant since all the patients were *de facto* in the ICU and under invasive MV (≥IVa) [21].

### Primary endpoint

The primary endpoint was the number of patients who had at least one surgical complication defined a priori (see above) that could be induced or worsened by prone position.

### Secondary endpoints

Secondary endpoints were the number of revision operations due to complication induced or worsened by prone position. Other secondary endpoints were effect of prone position on oxygenation duration of mechanical ventilation, ICU mortality and ICU length of stay.

### Statistical analysis

Statistical analysis was performed with GraphPad Prism 5 (GraphPad Software, San Diego, USA) and R version 3.1.2 (R Foundation for Statistical Computing, Vienna, Austria.). Categorical variables are described by their numbers and proportions and compared by the Fisher’s exact test. The normality of continuous variables was tested by the Kolmogorov–Smirnov test. Continuous variables of normal distribution are described by mean and standard deviation and compared by Student’s *t* test. Continuous variables of non-normal distribution are described by median and interquartile [25, 75%] range and compared by the Mann–Whitney test.

Primary endpoint was compared between the prone and supine groups using propensity score weighting to balance patient characteristics between the two groups. It was conducted in two stages. In the first stage, we performed a multivariate logistic regression to predict the probability of being in the prone group (i.e., the estimated propensity score (PS)), controlling for all the pre specified covariates (see above). In the second stage, we constructed logistic regression model to compare the risk of complication between prone and supine groups, using the inverse of the propensity score as a weight, targeting the average treatment effect in the whole population [22]. More precisely, a logistic model regressing the outcome with exposure (i.e., prone or supine group) as the only covariate was fitted, each subject being weighted according to its PS value, with a stabilized weight W equal to: W = pT/PS if subject is in the prone group, and (1 − pT)/(1 − PS) if subject is in the supine group, where pT is the is the overall probability of being in the prone group in the sample. Robust standard errors were used.

Variables considered for propensity score estimation were chosen based on empirical knowledge and included: age, weight, SAPS II, diabetes status, presence of a colonic stoma, of a small bowel stoma and of jejunostomy, use of catecholamines and delay from surgery to ICU hospitalization. No variable selection was performed. Balance on covariates between prone and supine groups was assessed and reported using absolute standardized differences (ASD) [23], and a sensitivity analysis with additional adjustment for covariates with ASD >10% after weighting was performed.

## Results

### Study population

**Fig. 1:**
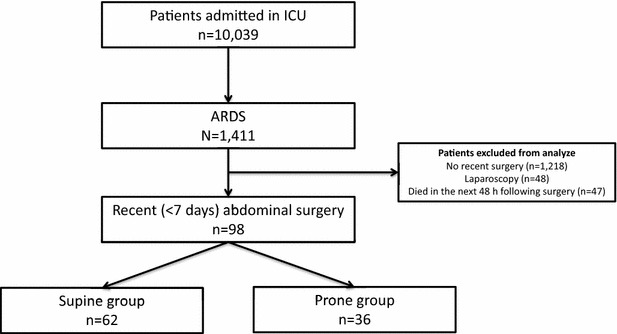
Patients flowchart of the 5-year period study

Among the 10,039 patients admitted to the participating ICU during the study period, 1411 had ARDS consistent with the Berlin definition [16]. Of these, 98 patients had undergone an abdominal surgery in the last 7 days (Fig. 1). Thirty-six patients (37%) had at least one prone position session and 62 (63%) remained supine.

 Table 1 shows that patients were severely ill as attested by high SAPS II scores and the requirement for catecholamine infusion at D0. Systemic hypertension and diabetes were more frequent in the supine group, and those patients had a higher SAPS II.

**Table 1:** Characteristics of patients

	Overall	Prone	Supine	p*
*n*	98	36	62	
Epidemiological data				
Age (years) (SD)	64 (18)	59 (19)	67 (17)	0.08
Male gender, *n* (%)	59 (60)	22 (61)	37 (60)	0.89
Weight (kg) (SD)	83 (24)	81 (26)	83 (24)	0.63
BMI (kg/m^2^) (SD)	31 (9)	30 (10)	31 (10)	0.77
COPD, *n* (%)	15 (15)	6 (17)	9 (15)	0.77
Ischemic heart disease, *n* (%)	15 (15)	4 (11)	11 (18)	0.38
Systemic hypertension, *n* (%)	60 (61)	13 (36)	47 (76)	*0.001*
Diabetes, *n* (%)	29 (30)	5 (14)	24 (39)	*0.01*
Characteristics of ICU severity				
SAPS II (SD) [17]	53 (17)	47 (15)	56 (17)	*0.02*
Septic shock (at D0), *n* (%)	75 (77)	26 (72)	49 (79)	0.44
Catecholamine infusion (at D0)	87 (89)	33 (92)	54 (87)	0.74
PaO_2_/FiO_2_ and ventilator settings at D0				
PaO_2_/FiO_2_ (mmHg) (SD)	91 (39)	74 (24)	101 (43)	*0.0005*
Plateau pressure (cm of water) (SD)	24 (6)	26 (4)	23 (7)	*0.02*
Tidal volume (ml) [IQR]	446 [400–497]	444 [400–500]	448 [400–496]	0.57
PEEP (cm of water) (SD)	11 (4)	13 (4)	10 (3)	*0.0001*
Adjunctive therapies (during ICU stay)				
Inhaled nitric oxide, *n* (%)	24 (24)	13 (36)	11 (18)	*0.04*
NMBA, *n* (%)	59 (60)	32 (89)	27 (43)	*0.0001*
Characteristics of abdominal surgery				
Emergent surgery, *n* (%)	79 (81)	30 (83)	49 (79)	0.60
Peritonitis, *n* (%)	41 (42)	14 (39)	27 (43)	0.65
Colonic resection, *n* (%)	22 (22)	4 (11)	18 (29)	*0.04*
Small bowel resection, *n* (%)	27 (28)	9 (25)	18 (29)	0.67
Gastric resection, *n* (%)	8 (8)	4 (11)	4 (6)	0.46
Esophageal resection, *n* (%)	7 (7)	3 (8)	4 (6)	0.71
Cholecystectomy *n* (%)	11 (11)	5 (14)	6 (10)	0.53
Partial hepatectomy, *n* (%)	3 (3)	2 (6)	1 (2)	0.28
Splenectomy, *n* (%)	1 (1)	0 (0)	1 (2)	1.00
Partial pancreatectomy, *n* (%)	3 (3)	0 (0)	3 (5)	0.30
Hysterectomy, *n* (%)	1 (1)	0 (0)	1 (2)	1.00
Parietal resection, *n* (%)	8 (8)	5 (14)	3 (5)	0.14
Cesarean, *n* (%)	3 (3)	2 (6)	1 (2)	0.55
HIPEC, *n* (%)	1 (1)	0 (0)	1 (2)	1.00
≥1 stoma, *n* (%)	34 (35)	10 (28)	24 (39)	0.27
Colonic stoma, *n* (%)	15 (15)	2 (6)	13 (21)	*0.04*
Small bowel stoma, *n* (%)	16 (16)	6 (17)	10 (16)	0.95
Jejunostomy, *n* (%)	5 (5)	2 (6)	3 (5)	1.00
Gastrostomy, *n* (%)	1 (1)	0 (0)	1 (2)	1.00
Open abdomen, *n* (%)	4 (4)	1 (3)	3 (5)	1.00

Italic values refer to a statistically significant *p*-value

*BMI* body mass index, *COPD* chronic obstructive pulmonary disease, SAPS II, *PEEP* positive end-expiratory pressure, *ICU* intensive care unit, *NMBA* neuromuscular blockade agent, *HIPEC* hyperthermic intraperitoneal chemotherapy, *SD* standard deviation, *IQR* interquartile [25, 75%]

* Prone versus supine

Respiratory failure at D0 was more severe in the prone group with a lower PaO_2_/FiO_2_ (74 ± 24 mmHg vs 101 ± 43 mmHg, *p* = 0.0005), a higher PEEP level (13 ± 3 vs 10 ± 3 cm of water, *p* = 0.0001), a higher plateau pressure (26 ± 4 vs 23 ± 7 cm of water, *p* = 0.02) and a more frequent use of adjunctive therapies.

Characteristics of abdominal surgery were similar in the two groups except for colonic resection and colonic stoma (more frequent in supine group). The delay between surgery and ICU hospitalization was 0 [0–1] days.

### Primary endpoint

Rate of surgical complications a priori induced or worsened by prone position did not differ between prone and supine groups [respectively: *n* = 14 (39%) vs *n* = 27 (44%); *p* = 0.65]. Details regarding these complications are summarized in Table 2.

**Table 2:** Postoperative surgical complications

	Prone *n* = 36	Supine *n* = 62	*p*
Scar dehiscence, *n* (%)	3 (8)	15 (24)	0.06
Abdominal compartment syndrome, *n* (%)	1 (3)	6 (10)	0.26
Stoma leakage, *n* (%)	1 (3)	13 (2)	1.00
Stoma necrosis, *n* (%)	3 (8)	3 (5)	0.67
Scar necrosis, *n* (%)	1 (3)	1 (2)	1.00
Wound infection, *n* (%)	6 (17)	5 (8)	0.20
Displacement of a peritoneal drainage system	0 (0)	1 (2)	1.00
Displacement of a biliary drainage system	0 (0)	1 (2)	1.00
Removal of a gastrostomy feeding tube	0 (0)	0 (0)	1.00
Removal of a jejunostomy feeding tube	1 (3)	0 (0)	0.37
Digestive fistula	3 (8)	11 (18)	0.24

NB: one patient could have several complications. This explains that the total (19 for prone group and 56 for supine group) may be different than the number of primary endpoint (define as “at least one surgical complication”)

After propensity score application, there was still no significant difference between the two groups (OR 0.72 [0.26–2.02], *p* = 0.54). Since an imbalance was detected after propensity score weighting for variable “colonic stoma” (Additional file 1: Figure 1E), an analysis adjusting for this covariate was also performed, with unchanged results (data not shown).

### Secondary endpoint (Table 3)

**Fig. 2:**
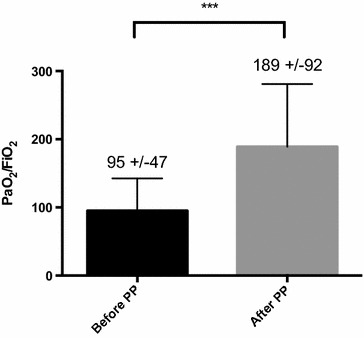
*Bar* graph representing changes in mean PaO_2_/FiO_2_ before and after first prone position session. There was a significant increase in this ratio after the first session of prone (*p* < 0.0001). PaO_2_/FiO_2_ before PP: measure on the last arterial blood gas before first prone session; PaO_2_/FiO_2_ after PP: measure on the first arterial blood gas during the first prone position session. *PP* prone position

 Twenty-one (58%) patients were turned prone in the first 48 h following the surgery (median time between surgery and first prone session 2 [1–4] days). The median number of prone session was 1 [1–2] (1 session: 19 patients, 2 sessions: nine patients, 3 sessions: six patients, 4 sessions: one patient, 5 sessions: one patient). The duration of the first and second session were, respectively, 15.8 (±10.4) and 19.2 (±10.3) hours. PaO_2_/FiO_2_ ratio improves dramatically after the first prone session (Fig. 2).

**Table 3:** Primary and secondary endpoints

	Overall	Prone	Supine	*p*
Primary endpoint^a^, *n* (%)	41 (42)	14 (39)	27 (44)	0.65
Revision surgery^b^, *n* (%)	17 (17)	3 (8)	14 (23)	0.10
All revision surgery^c^	35 (36)	8 (22)	26 (42)	0.10
Duration of MV	10 [6–17]	9.5 [6–21]	11 [6–15]	0.72
ICU length of stay	13 [7–22]	13 [8–24]	13 [6–21]	0.77
ICU mortality^d^, *n* (%)	43 (44)	15 (42)	28 (45)	0.43

*MV* mechanical ventilation, *ICU* intensive care unit

^a^At least one surgical complication that could be induced or worsened by prone position

^b^For primary endpoint

^c^Several patients had more than one revision surgery

^d^Five patients died in the first 48 h (two in the prone group and three in the supine group)

During the first 2 h of the first prone session, 26 (72%) patients were hemodynamically stable, six (17%) experienced hemodynamic worsening and four (11%) experienced hemodynamic improvement.

Rate of revision surgery did not differ between the two groups. Duration of MV, ICU length of stay and ICU mortality were also not different (Table 3).

## Discussion

This is the first retrospective multicentre study evaluating safety and efficacy of prone position for severe post-abdominal surgery ARDS patients. We found that early postoperative prone position was not associated with increased local or surgical complications and that oxygenation significantly improved after one session of proning. These results were found in a varied population of patients, in three distinct hospitals, which give credits to their generalizability. They may have an immediate and significant impact on patient outcome, given the recent demonstration of the strong survival benefit of prone position during ARDS [11].

Despite large RCT in this context [5–8, 11], data regarding post-abdominal surgery patients are missing. Indeed, although post-abdominal surgery is not stated as a contraindication to prone position, it is difficult to extract specific figures regarding this population from these studies. This is either due in part to the fact that the precise number of patients with post-abdominal surgery is not provided [5, 7, 8] or because the definition of postoperative acute respiratory failure encompasses patients with very low risks of surgical complications (endoscopic procedures and interventional radiological procedures) (6, 11). The lack of such available data led us to investigate the safety of prone position in abdominal postoperative ARDS patients. Our results could provide clinicians with answers to the following questions: is the prone position associated with a greater rate of surgical complications? Does it yet improve oxygenation?

We deliberately chose not to investigate other known adverse events related to prone position because these complications are not more frequent in patients turned prone [10].

Data on the rate of surgical complications are scarce. Offner et al. [14] described four patients with multisystem trauma placed prone after midline abdominal incisions for exploratory laparotomy. Among them, one experienced wound dehiscence. Authors suggested that careful consideration was required before turning prone this subset of patients. However, number of patients studied was very small and no comparison with patients kept supine was made, preventing any definite answer to the question. On the opposite, our results offer a clear answer: We found no increase in the number of complications even after using a propensity score. This result stemmed from an exhaustive analysis of the patients’ charts and medical files, using an a priori a list of surgical complications possibly related to prone position, established in collaborations with surgeons at the participating centers and confronted to an analysis of the literature on the subject.

Regarding oxygenation, in this patient population, little is available in the literature. In a small retrospective study, Davis et al. [24] described trauma and surgical patients with acute lung injury and ARDS and questioned the benefit of prone position. Others found that oxygenation was improved by the prone position, which suggests the effectiveness of the technique in terms of oxygenation. However, numbers of patient were small and data regarding complications and more specifically surgical complications were not reported.

Our results confirm that in patients with postsurgical ARDS, prone positioning provides a clear benefit in terms of oxygenation. Compared to results from the large RCTs, we found a comparable if not greater improvement in PaO_2_/FiO_2_ ratio. Gattinoni et al. [5] and Guerin et al. [11] report a PaO2/FIO2 increase after the first prone session of approximately 60 and 50 mmHg, respectively. Ours was almost 100 mmHg, which confirms and extends that patients with postsurgical ARDS are particularly responsive to prone position.

### Strengths and weaknesses

Because Guerin et al. unambiguously demonstrated the clear benefit in terms of survival of prone position in a large population of very diverse etiologies of ARDS, there is obviously no case for another RCT in the specific setting of post-abdominal surgery (6). Indeed, we observed that 40% of patients had at least one surgical complication potentially related to position in the present study. To test the non-inferiority of prone position against supine position with a proper RCT, approximately 2400 subjects would be necessary to obtain a power of 80%, with a non-inferiority margin of 5%, and a type I error rate of 5%. It would take years to complete such a RCT. Given the small numbers of patients concerned by this condition, we felt that a retrospective study could help address our question. This choice has by design some limitations (including possible confounding effect, undisclosed bias in the decision of being or not turned prone). However, these were counterbalanced by the multicenter design of our study and the number of patients included which constitutes to date the largest study on the subset of postsurgical patients. Additionally, our database is part of a larger network used by many ICUs in Paris and its suburbs called CUB-Réa, and several publications have already been made with the data extracted from this database, so as to prove its efficacy and reliability [25, 26]. Although certain specifics of the prone sessions could not be traced in the records (e.g., staff required to turn the patient prone, number and location of pillows used), protocol used in the three ICUs was very similar and included placement of pillows under the thorax and pelvis in order to limit abdominal pressure [27]. We acknowledge the fact that the number of prone sessions was lower than in PROSEVA. The possibility that a greater number of prone sessions could be associated with an increased risk of surgical complication cannot be ruled out. However, intuitively, one can reason that the risk of complications specifically related to the surgery is greater in the early days after surgery. Because more than half the patients were turned prone within the first 48 h after surgery, we believe this limits the risks of having missed some complications because of insufficient number of prone sessions.

Baseline characteristics differed slightly between the two groups: Supine patients were older. This difference may impact related variables such as arterial hypertension, diabetes and SAPS II score. Nonetheless, the use of a propensity score analysis that takes into account these differences confirmed the initial findings.

Despite our conclusive results, the decision to turn a post-abdominal surgery patient prone should be taken on a case-by-case basis after discussion between the surgeons and the intensivists. Several issues could restrict use of prone position, such as multiple intra-hospital transport for CT scan, need for frequent revision surgery or presence of a Mikulicz drainage system. Nonetheless, we believe none of the above represents an absolute contraindication, and all are outweighed in case of life-threatening hypoxemia.

To conclude, our results confirm the effectiveness of prone positioning in terms of oxygenation in ARDS after abdominal surgery without significant increase in surgical complications and no effect on the need for surgical revisions. Hence, if necessary, our results suggest that clinicians should not refrain from proning patients with post-abdominal surgery ARDS.

## Additional file

**Additional file 1**. Figure 1E: Imbalances between prone and supine groups before and after propensity score weighting. This figure is a graphical representation of absolute standardized differences, showing imbalances of patients’ baseline characteristics between prone and supine groups before and after propensity score weighting. A standardized difference <10% indicates excellent covariate balance. Red circle symbol: without weighting. Blue circle symbol: using the inverse of the propensity score as a weight.

## Abbreviations

ARDS: acute respiratory distress syndrome
VILI: ventilator-induced lung injury
RCT: randomized controlled trials
STROBE: Strengthening the Reporting of Observational Studies in Epidemiology
ICU: intensive care units
BMI: body mass index

## References

[1] Piehl MA, Brown RS (1976). Use of extreme position changes in acute respiratory failure. Crit Care Med.

[2] Gattinoni L, Carlesso E, Taccone P (2010). Prone positioning improves survival in severe ARDS: a pathophysiologic review and individual patient meta-analysis. Minerva Anestesiol.

[3] Valenza F, Guglielmi M, Maffioletti M (2005). Prone position delays the progression of ventilator-induced lung injury in rats: does lung strain distribution play a role?. Crit Care Med.

[4] Broccard A, Shapiro RS, Schmitz LL (2000). Prone positioning attenuates and redistributes ventilator-induced lung injury in dogs. Crit Care Med.

[5] Gattinoni L, Tognoni G, Pesenti A (2001). Prone-Supine Study Group: effect of prone positioning on the survival of patients with acute respiratory failure. N Engl J Med.

[6] Guerin C, Gaillard S, Lemasson S (2004). Effects of systematic prone positioning in hypoxemic acute respiratory failure: a randomized controlled trial. JAMA.

[7] Mancebo J, Fernández R, Blanch L (2006). A multicenter trial of prolonged prone ventilation in severe acute respiratory distress syndrome. Am J Respir Crit Care Med.

[8] Taccone P, Pesenti A, Latini R (2009). Prone-Supine II Study Group: prone positioning in patients with moderate and severe acute respiratory distress syndrome: a randomized controlled trial. JAMA.

[9] Abroug F, Ouanes-Besbes L, Elatrous S (2008). The effect of prone positioning in acute respiratory distress syndrome or acute lung injury: a meta-analysis. Areas of uncertainty and recommendations for research. Intensive Care Med.

[10] Sud S, Taccone P, Polli F (2010). Prone ventilation reduces mortality in patients with acute respiratory failure and severe hypoxemia: systematic review and meta-analysis. Intensive Care Med.

[11] Guerin C, Reignier J, Richard J-C (2013). Prone positioning in severe acute respiratory distress syndrome. N Engl J Med.

[12] Gattinoni L, Taccone P, Carlesso E (2013). Prone position in acute respiratory distress syndrome. Rationale, indications, and limits. Am J Respir Crit Care Med.

[13] Bellani G, Laffey JG, Pham T (2016). LUNG SAFE Investigators, ESICM Trials Group: epidemiology, patterns of care, and mortality for patients with acute respiratory distress syndrome in Intensive Care Units in 50 countries. JAMA.

[14] Offner PJ, Haenel JB, Moore EE (2000). Complications of prone ventilation in patients with multisystem trauma with fulminant acute respiratory distress syndrome. J Trauma.

[15] von Elm E, Altman DG, Egger M (2007). STROBE Initiative: the Strengthening the Reporting of Observational Studies in Epidemiology (STROBE) statement: guidelines for reporting observational studies. Ann Intern Med.

[16] Definition Task Force ARDS, Ranieri VM, Rubenfeld GD, Thompson BT (2012). Acute respiratory distress syndrome: the Berlin definition. JAMA.

[17] Le Gall JR, Lemeshow S, Saulnier F (1993). A new Simplified Acute Physiology Score (SAPS II) based on a European/North American multicenter study. JAMA.

[18] Bone RC, Balk RA, Cerra FB (1992). Definitions for sepsis and organ failure and guidelines for the use of innovative therapies in sepsis. The ACCP/SCCM Consensus Conference Committee. American College of Chest Physicians/Society of Critical Care Medicine. Chest.

[19] Calandra T, Cohen J (2005). International sepsis forum definition of infection in the ICU consensus conference: the international sepsis forum consensus conference on definitions of infection in the intensive care unit. Crit Care Med.

[20] De Waele JJ, De Laet I, Kirkpatrick AW (2011). Intra-abdominal hypertension and abdominal compartment syndrome. Am J Kidney Dis Off J Natl Kidney Found.

[21] Clavien PA, Barkun J, de Oliveira ML (2009). The Clavien-Dindo classification of surgical complications: five-year experience. Ann Surg.

[22] Austin PC (2011). A tutorial and case study in propensity score analysis: an application to estimating the effect of in-hospital smoking cessation counseling on mortality. Multivar Behav Res.

[23] Ali MS, Groenwold RHH, Belitser SV (2015). Reporting of covariate selection and balance assessment in propensity score analysis is suboptimal: a systematic review. J Clin Epidemiol.

[24] Davis JW, Lemaster DM, Moore EC (2007). Prone ventilation in trauma or surgical patients with acute lung injury and adult respiratory distress syndrome: is it beneficial?. J Trauma.

[25] Zuber B, Tran T-C, Aegerter P (2012). CUB-Réa network: impact of case volume on survival of septic shock in patients with malignancies. Crit Care Med.

[26] Annane D, Aegerter P, Jars-Guincestre MC (2003). CUB-Réa network: current epidemiology of septic shock: the CUB-Réa network. Am J Respir Crit Care Med.

[27] Kirkpatrick AW, Pelosi P, De Waele JJ (2010). Clinical review: intra-abdominal hypertension: does it influence the physiology of prone ventilation?. Crit Care.

