# Supplementary material for: Prone positioning in acute respiratory distress syndrome after abdominal surgery: a multicenter retrospective study: SAPRONADONF (Study of Ards and PRONe position After abDOmiNal surgery in France)
Source: Ann Intensive Care. 2017 Feb 24;7:21. doi: 10.1186/s13613-017-0235-z (PMC5325801; doi:10.1186/s13613-017-0235-z)

**Supplemental Digital Content**

**Figure 1E:** Imbalances between prone and supine groups before and after propensity score weighting.

This figure is a graphical representation of absolute standardized differences, showing imbalances of patients’ baseline characteristics between prone and supine groups before and after propensity score weighting. A standardized difference <10% indicates excellent covariate balance. Red circle symbol: without weighting. Blue circle symbol: using the inverse of the propensity score as a weight.


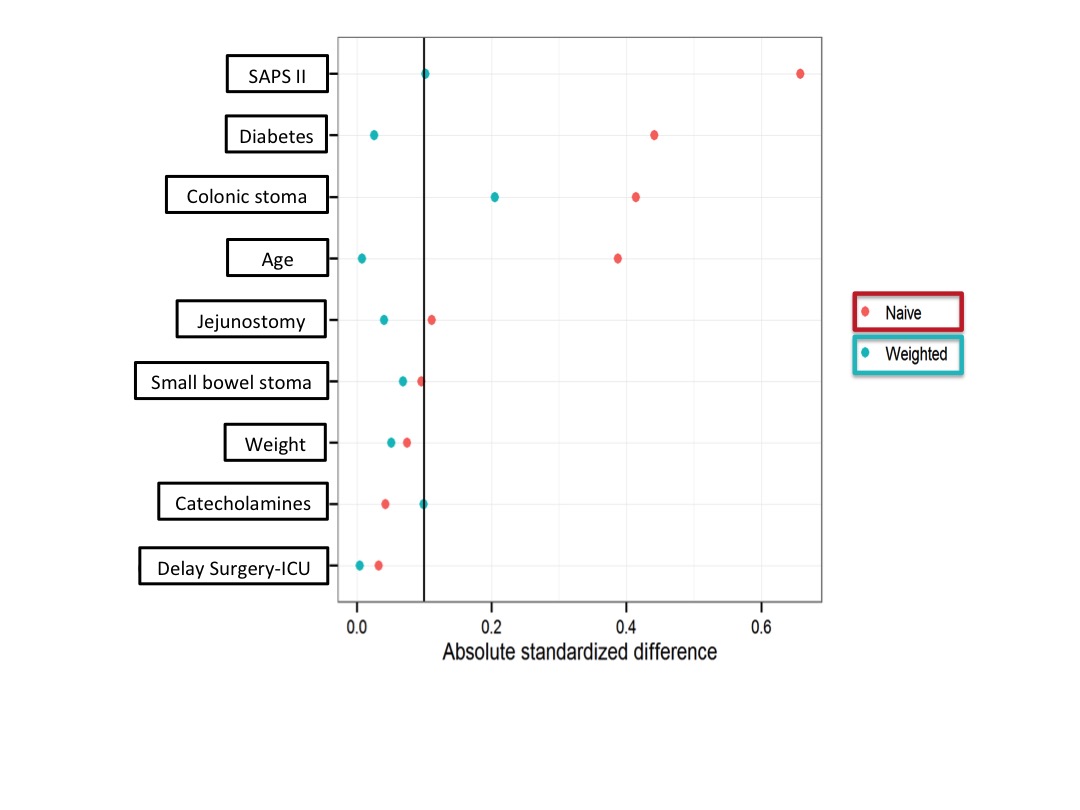

Supplement: Supplementary file 1 — Additional file 1. Figure 1E: Imbalances between prone and supine groups before and after propensity score weighting. This figure is a graphical representation of absolute standardized differences, showing imbalances of patients’ baseline characteristics between prone and supine groups before and after propensity score weighting. A standardized difference <10% indicates excellent covariate balance. Red circle symbol: without weighting. Blue circle symbol: using the inverse of the propensity score as a weight. [file 13613_2017_235_MOESM1_ESM.docx]
